# Economic uncertainty and population health: insights from emerging markets and developing countries

**DOI:** 10.3389/fpubh.2023.1292236

**Published:** 2023-10-25

**Authors:** Lin Liu

**Affiliations:** Department of Accounting, School of Economics and Management, Nantong University, Nantong, China

**Keywords:** economic uncertainty, health, emerging markets, developing countries, Bayesian panel vector autoregressive model JEL I150, C59, E66

## Abstract

This study employs a Bayesian panel vector autoregressive model to examine the impact of economic uncertainty on public health, using an annual, country-level panel dataset of 103 emerging markets and developing countries spanning the years 1995 through 2019. The results from the full sample suggest that the immediate effects of heightened economic uncertainty on health are marginal, yet it may engender prolonged life expectancy and lowered mortality rates. The analysis unveils considerable heterogeneities among various country classifications. The health-enhancing effects of economic uncertainty are predominantly discernible in emerging markets, low-income and upper-middle-income countries. Additionally, a diminution in suicide rates, attributed to escalated economic uncertainty, is uniquely detected in upper-middle-income countries. Furthermore, economic growth and healthcare expenditure emerge as paramount determinants in bolstering overall population health, particularly in lower-middle-income countries. The detrimental effect of environmental pollution on health is more pronounced in emerging markets and middle-income nations. Excluding high-income countries, it is essential to emphasize the beneficial health outcomes resulting from financial development and globalization, as well as the deleterious effects of environmental pollution. Lastly, several policy implications aligned with the findings are outlined, providing a roadmap for decision-makers in these diverse economies to promote better health outcomes.

## 1. Introduction

As reported by the World Health Organization (1), significant strides in global human development, particularly in health status, were observed before 2020. The worldwide average life expectancy at birth, a well-recognized indicator of population health, rose from 52.58 years in 1960 to 72.74 years in 2019. Fascinatingly, emerging markets and developing countries experienced a remarkable life extension of approximately 25 years. These advancements in health primarily mirror the progress made in reducing child mortality rates and combating infectious diseases (2), subsequently sparking burgeoning interests in investigating the impact of economic activity on population health, particularly within emerging markets and developing countries.

Since late 2019, the world has been grappling with the COVID-19 pandemic for nearly 3 years. This period has seen two striking phenomena: the pandemic has not only wheeled up economic uncertainty to unprecedented levels but also posed a profound threat to public health. The World Uncertainty Index (WUI) and the Global Economic Policy Uncertainty Index (GEPU), two prominent measures of economic uncertainty developed by Ahir et al. (3) and Baker et al. (4), respectively, reached record highs in early 2020, coinciding with the global spread of the pandemic. Concurrently, COVID-19 became a leading cause of worldwide death and has remarkably shortened life expectancy in many countries (1). Increased anxiety, depression, and other harmful health behaviors have been monitored during the COVID-19 pandemic (5–7). Besides its direct epidemiological damage, the pandemic could indirectly impact population health through heightened economic uncertainty, which is known to sway economic activities.

While previous studies have substantiated the roles of the macroeconomy in population health, the effects of economic uncertainty-a principal source of economic disturbances (8)-on population health are not fully understood. Recent studies have attempted to probe the aftermath of economic uncertainty on mental health indicators, but a comprehensive understanding of its role in public health remains elusive. For instance, the evidence by Antonakakis and Gupta (9), Vandoros et al. (10), Abdou et al. (11), Abdou et al. (12), and Claveria (13) suggests that spikes in economic uncertainty may contribute to human mental disorders and suicides. Kalcheva et al. (14) argue that economic uncertainty can lead to unhealthy lifestyle habits. Furthermore, Vandoros et al. (15) populate that excessive mental strain and poor lifestyle habits induced by heightened economic uncertainty may increase the occurrence of accidents.

However, the health outcomes of economic uncertainty are not unequivocal. On the one hand, increased economic uncertainty would diminish household income and exacerbate income unpredictability, significantly undermining public well-being and daily-life happiness (16). Anxiety, worry, and even depression stemming from economic uncertainty might drive individuals to adopt lousy lifestyle choices (14), further impairing their physical health. Additionally, under heightened economic uncertainty, households and governments might be motivated to cut healthcare spending, which could negatively impact public health. On the other hand, following spikes in economic uncertainty, investment and industrial production declines could potentially reduce the incidence of industrial accidents and pollutant emissions, yielding beneficial repercussions for public health. Crucially, whether and how economic uncertainty impacts public health is still unclear in the literature related to health topics. Against these backdrops, this paper aims to bridge this gap in the literature by exploring the general health outcomes of economic uncertainty through a cross-country lens.

Notably, I emphasize emerging markets and developing countries (hereafter EMDCs). The reasons are threefold. First, compared to developed countries, these nations are more susceptible to internal and external shocks (17) because of imperfect economic and financial systems. Second, uncertainty shocks may trigger more substantial macroeconomic declines in EMDCs (18), resulting in more significant health deterioration consequentially due to inadequate medication and modern medical infrastructure in these countries (19). Third, given that the epicenter of the COVID-19 pandemic has been dramatically shifting to EMDCs (1), exploring the health impact of uncertainty shocks in these countries can help governments better understand the determinants of population health, particularly health dynamics under elevating economic uncertainty, and subsequently narrow the health gap among countries.

To my knowledge, this study presents the first comprehensive examination of the linkages between economic uncertainty and public health. Understanding the endogenous relationships between population health and the macroeconomy is essential as they are inextricably connected. Moreover, the macroeconomy predominantly functions as a dynamic system with interacting and interdependent economic variables. As such, addressing the features of endogeneity is critical when examining the connections between health and the macroeconomy. In this study, I first disentangle the theoretical linkages between economic uncertainty and population health by discussing four channels through which economic uncertainty could affect population health. Given the theoretical analysis and recognizing the interconnectedness of population health and the macroeconomy, I then apply the Bayesian panel vector autoregressive (BPVAR) model proposed by Beetsma et al. (20) to a well-assembled annual panel dataset of 103 EMDCs from 1995 to 2019 to implement the empirical investigation.

The empirical findings from the whole sample show that, in EMDCs, the effects of heightened economic uncertainty on public health are relatively marginal and less perceptible in the short term. A transient beneficial impact on health, evident in prolonging life expectancy and reducing aggregate mortality rates, emerges from rising economic uncertainty. However, surges in economic uncertainty may considerably curtail life expectancy and raise mortality rates, while paradoxically lowering suicide rates in the long term. This finding remains robust across different model specifications, including VAR lag length and model dimensionality. Economic growth and healthcare expenditure emerge as salient factors augmenting overall population health in EMDCs, with additional verification of the health-promoting influences of financial development and globalization. The detrimental health effects induced by environmental pollution are also accentuated.

Further, the subsample analysis uncovers significant heterogeneities among distinct categorizations of the sample countries, providing additional empirical evidence on uncertainty-health relations. Concentrating on life expectancy, aggregate mortality, and child mortality, the results disclose that the health-enhancing effects of economic uncertainty are more conspicuous in emerging markets, low-income and upper-middle-income countries. Notably, surges in economic uncertainty may precipitate elevated child mortality rates in high-income and lower-middle-income countries. The positive impacts of healthcare expenditure on health are reaffirmed, with more noticeable health gains from healthcare expenditure in developing, low-income, and lower-middle-income countries. The beneficial health outcomes associated with economic growth are more emphatic in lower-middle-income countries but are fleeting and negligible in low-income countries.

Conversely, high-income countries may experience health deterioration alongside economic growth. The pernicious effects of environmental pollution on health are solely discernible in emerging markets, lower-middle-income, and upper-middle-income countries. Financial development has no apparent impact on health in developing and high-income countries, while the significant salubrious effects of globalization are observed in all nation classifications except high-income countries. A substantial decline in suicide rates following rising economic uncertainty shocks is exclusively evident in upper-middle-income countries. Increased economic growth and healthcare expenditure are succeeded by escalating suicide rates in emerging markets, developing countries, and all income countries, except low-income countries.

The remainder of this paper is organized as follows. Section 2 offers a review of recent studies relevant to this research. Section 3 discusses the theoretical channels through which economic uncertainty could impact population health and outlines the empirical methodology utilized in this study. Section 4 presents the empirical analysis, including the results of baseline models, the subsequent robustness checks, and heterogeneity analysis. The final section concludes the whole paper, underscores the policy implications, and discusses the limitations of this study and directions for future research.

## 2. Literature review

The influence of economic uncertainty, a complex variable to quantify, on population health, has only recently been scrutinized in a handful of studies. Individuals dwelling in environments rife with high levels of uncertainty may experience a range of psychological distress, such as anxiety, worry, and even mental disorders, particularly if they exhibit high intolerance towards uncertainty (21). Considering this analysis, several efforts have been dedicated to evaluating the mental repercussions of shocks in economic uncertainty. Yagil and Cohen (22) further demonstrate that financial uncertainty can trigger anxiety during the COVID-19 pandemic, based on survey data gathered from Israeli citizens.

Given that mental disorders are a primary trigger for suicide, several studies have explored the impact of EPU on suicide mortality. Antonakakis and Gupta (9), Abdou et al. (11), Abdou et al. (12) examine US subpopulations in terms of age and gender, while Vandoros et al. (10) provide insights for England and Wales. Claveria (13) extends this analysis to 183 economies. Antonakakis and Gupta (9) conclude that rises in economic policy uncertainty (EPU) cause higher suicide mortality among the youngest and oldest males, while US females seem to show a minimal response to such shocks. Moreover, Abdou et al. (11) find a significant impact of financial uncertainty on U.S. suicide rates among the youngest males and middle-aged females. Subsequently study by Abdou et al. (12) observes significant asymmetric effects of EPU on U.S. suicide rates in middle-aged males and older females, with only extreme unexpected surges in EPU triggering suicide crises within these demographics. The gender-dependent aspect of these findings is echoed by Vandoros et al. (10), who find that increases in EPU result in a rise in suicide rates within 2 days. However, the results presented in Claveria (13) are mixed and inconsistent.

Another strand of the literature investigates the outcomes of economic uncertainty shocks on physical health. Hikes in economic uncertainty may impose psychological pressures on individuals, promoting them to adopt unhealthy lifestyle behaviors. Kalcheva et al. (14) report a positive correlation between EPU and the tendency towards unhealthy lifestyle habits, such as increased alcohol consumption and smoking. Additionally, excessive mental stress and unhealthy lifestyle habits may lead to a higher incidence of accidents. Vandoros et al. (15), utilizing daily data from the UK, identify sudden jumps in motor vehicle collisions following spikes in UK EPU.

In summary, prior research has made significant strides in examining the relationship between economic uncertainty and population health outcomes, such as mental health, suicide mortality, and unhealthy behaviors. Preliminary evidence suggests economic uncertainty may harm mental health and increase risky behaviors. However, exploring how economic uncertainty might impact public health needs to be more thorough and merits further investigation. Moreover, most previous studies have focused on a single economy, leaving global evidence needing more attention, particularly in emerging markets and developing countries. To bridge these gaps, this paper aims to concentrate on emerging markets and developing countries, evaluating the impact of economic uncertainty on general public health.

## 3. Theoretical background and empirical methodology

### 3.1. Theoretical background

The impact of economic uncertainty on population health is manifold, operating through both direct and indirect channels. The direct channel primarily implicates mental health, often called the psychological channel. Concurrently, three indirect channels are mediated through the effects of economic uncertainty on health-related economic indicators, specifically economic growth, healthcare expenditure, and environmental pollution (quality).

#### 3.1.1. Psychological channel

Economic uncertainty can provoke widespread public apprehension regarding economic stability, potentially inciting anxiety about dwindling income, the threat of unemployment, and even the emergence of mental disorders or suicidal tendencies (10–12). While mental health deterioration is a grave concern, it is merely one facet of population health and does not inevitably portend a decline in overall health conditions.

#### 3.1.2. Economic growth channel

Economic output and population health share a reciprocal relationship (23, 24). Economic growth boosts *per capita* income, enhances national purchasing power for goods and services, and improves living standards. Furthermore, economic advancements engender superior and high-quality healthcare and medical services, culminating in improved population health. Although the health-growth literature is not entirely conclusive, the preponderance of studies substantiates the positive ramifications of economic development on health, see O’Donoghue et al. (25), Subramanian et al. (26), Tapia Granados and Ionides (27), Renton et al. (28), Morgado (29), Cole (30), amongst others. Recent studies by Wang (31), Breuer (32), and Wang et al. (33) assert a synchronization of mortality with unemployment across various countries. Therefore, while economic growth is vital for health, contractions in economic growth driven by heightened economic uncertainty could bring adverse health consequences.

#### 3.1.3. Healthcare expenditure channel

Within the realm of health economics, health is conceptualized as an output of healthcare services, with consumers deriving utility from both consumption and health outcomes (34). Greater healthcare expenditure generally promotes health (35). However, healthcare expenditure often hinges on economic growth (36). Governments may curtail public healthcare expenditure in response to declined economic output following a surge in economic uncertainty (37, 38). Also, according to the absolute income hypothesis (39), households tend to reduce consumption in the face of declining income. Existing literature reveals that economic uncertainty can trigger precautionary incentives among families. As healthcare expenditure constitutes a significant segment of household consumption, potential income reductions due to escalating economic uncertainty could compel households to cut private healthcare expenditure, thereby hampering better health outcomes.

#### 3.1.4. Environmental pollution (quality) channel

Environmental pollution and degradation are associated with economic growth, especially in developing countries (40–42). Pollution can exert a direct, deleterious effect on human health (43–45). Hence, environmental improvements and concomitant health benefits might be expected during economic downturns. From this perspective, a surge in economic uncertainty may reduce investment activities and pollution, potentially facilitating public health enhancements. However, exacerbated economic uncertainty could also incentivize governments to ease environmental regulations to counter potential economic recessions, encouraging firms to augment pollutant emissions (46), which could detrimentally affect public health.

### 3.2. Empirical methodology

In this study, I utilize a panel vector autoregressive model (hereafter referred to as panel VAR) to implement empirical investigation. To preserve model parsimony, the panel VAR model comprises five endogenous variables: population health indicator(s), economic uncertainty, economic growth, healthcare expenditure, and environmental pollution. Notably, given that the characteristics of the sample data-short T and large N-I abstain from incorporating cross-sectional interactions and parameter variations across both time and units. Adopting such a complex model would make the estimation overly complicated. Instead, I employ the more restrictive Bayesian panel VAR model proposed by Beetsma et al. (20). Still, I continue to assess the heterogeneous effects across countries by dividing the samples according to country characteristics.

Compactly, the annual panel VAR with fixed effects can be expressed in a concise vector form as follows:


(1) $$Y_{it}=\alpha_{i}+\beta_{i}t,+\Sigma_{j=1}^{P}A_{j}Y_{it-j}+u_{it},$$

where *i* denotes the country and *t* represents the year, $Y_{it}$ is a vector of endogenous variables of interest, $\alpha_{i}$ measures country-fixed effects, $\beta_{i}t$ is the term reflecting country-specific linear trend effect, and $u_{it}$ is a vector of residuals assumed to follow a normal distribution with zero-mean and constant variance Ω. The lag length chosen for the VAR process is denoted by *P*, and $A_{j}$ is the matrix of coefficients of the *j*-th lag of the endogenous variables.

As highlighted by previous studies, economic conditions and population health may have simultaneous relationships, which warrant consideration in the empirical framework. To this end, I introduce a lower triangular matrix $A_{0}$ to capture the contemporaneous impact and identify the structural shocks by implementing a Cholesky decomposition of the residual covariance matrix Ω, i.e.,


(2) $$\Omega =A_{0}A_{0}^{\prime }.$$

In the baseline model, variables in $Y_{it}$ are ordered as follows,


(3) $$Y_{it}={\left(\begin{array}{l} \text{economic}\_{\text{uncertainty}}_{it},\text{economic}\_{\text{growth}}_{it},\\ \text{enviornmental}\_{\text{quality}}_{it},\text{healthcare}\_{\text{expenditure}}_{it},\\ \text{health}\_{\text{indicator}}_{it} \end{array}\right)}^{\prime }$$

The economic uncertainty indicator is ordered first since it can be predominantly regarded as a variable with low degrees of endogeneity to economic activity (47). In the baseline model, population health is placed last, reflecting the immediate impact of economic conditions on health, with the inverse less likely. Following Beetsma et al. (20), I estimate the models using a Markov chain Monte Carlo (MCMC) Gibbs sampling algorithm.

Consider a dataset that spans over *T* years and across *N* countries. The designed MCMC Gibbs algorithm operates as follows^1^:

The model parameters to be estimated are stacked in a vector *B*, where $B=vec\left(\left[\alpha_{i},\beta_{i},A_{j}\right]\right)$, and the regressors in Eq. (1) are defined as *X*. Hence, given the prior distribution, the posterior distribution is inferred sequentially from two conditional posterior distributions, G(B|Ω) and G(Ω|B).

G(B|Ω) rests on a normal density $N\left(B^{*},\Omega ⊗{\left(X^{*}{\;}^{\prime }X^{*}\right)}^{-1}\right)$, where $B^{*}={\left(X^{*}{\;}^{\prime }X^{*}\right)}^{-1}\left(X^{*}{\;}^{\prime }Y^{*}\right)$. Here, $X^{*}=\left(\begin{array}{c} X\\ X_{D} \end{array}\right)$ and $Y^{*}=\left(\begin{array}{c} Y\\ Y_{D} \end{array}\right)$ represent the stacked vector of actual data with dummy observations. G(Ω|B) adheres to an inverse Wishart distribution $IW\left(S^{*},\;NT+T_{D}-K\right)$, where $S^{*}={\left(Y-XB\right)}^{\prime }\left(Y-XB\right)$. $T_{D}$ is the size of dummy observations and K stands for the number of regressors in each equation in the VAR system. The algorithm continues to iterate until the draws converge.

For setting the prior, two $T_{D}$ dummy observations are defined as $Y_{D}=\left(\begin{array}{c} Y_{D1}\\ Y_{D2} \end{array}\right)$ and $X_{D}=\left(\begin{array}{c} X_{D1}\\ X_{D2} \end{array}\right)$, where $Y_{D1}=\left(\begin{array}{c} diag\left(\gamma \sigma_{1},\ldots ,\gamma \sigma_{N}\right)\\ \begin{array}{c} \begin{array}{c} 0_{N\left(P-1\right)\times N}\\ \ldots \end{array}\\ \begin{array}{c} diag\left(\sigma_{1},\ldots ,\sigma_{N}\right)\\ \begin{array}{c} \ldots \\ 0_{1\times N} \end{array} \end{array} \end{array} \end{array}\right)$, $X_{D1}=\left(\begin{array}{cc} \frac{diag\left(1,\ldots ,P\right)\times diag\left(\sigma_{1},\ldots ,\sigma_{N}\right)}{\tau } & 0_{NP\times 1}\\ \begin{array}{c} 0_{N\times NP}\\ \begin{array}{c} \ldots \\ 0_{1\times NP} \end{array} \end{array} & \begin{array}{c} 0_{N\times 1}\\ \begin{array}{c} \ldots \\ c\times I_{ex} \end{array} \end{array} \end{array}\right)$, $Y_{D2}=\frac{diag\left(\gamma \mu 1,\ldots ,\gamma \mu_{N}\right)}{\lambda }$, $X_{D2}=\left(\frac{\left(I_{1\times P}\right)⊗diag\left(\gamma \mu_{1},\ldots ,\gamma \mu_{N}\right)}{\lambda }\;\;0_{N\times 1}\right)$, γ symbolizes the mean of the prior, $\sigma_{i}$ is scaling factor set by initial AR(1) regressions, τ signifies the tightness of the prior of the autoregressive coefficients, and c controls the prior for the exogenous regressors in the equation (the number of which is denoted as *ex*). The prior on the VAR coefficients and error covariance is implemented through $Y_{D1}$ and $X_{D1}$, while $Y_{D2}$ and $X_{D2}$ are used for the prior on the sum of coefficients on the lagged endogenous variables. Following Beetsma et al. (20), in the following empirical study, the hyper-parameters are set to reflect a loose prior belief with τ=1, c=0.001, λ=10, respectively.

## 4. Data and results

### 4.1. Data

This study draws upon an annual, country-level dataset encompassing 103 emerging markets and developing countries from 1995 to 2019. The dataset selection is primarily dictated by data availability,^2^ with the specifics of the countries in the sample and their corresponding characteristics provided in Table A1 in Appendix A.

In evaluating economic uncertainty, I use an innovative metric known as the World Uncertainty Index (WUI), introduced by Ahir et al. (3). Employing text-mining methodologies, the WUI effectively monitors uncertainties stemming from economic and political events across 143 countries. The WUI uniquely extracts information from a single, consistent source-the economist intelligence unit country reports-thereby facilitating cross-country comparisons of uncertainty levels and their subsequent effects. Consequently, aligning with the studies of Gozgor et al. (48), Afzali et al. (49), and Fang et al. (50), I adopt the WUI as the measure of economic uncertainty. The quarterly index retrieved from *worlduncertiantyindex.com* is converted into an annual average for each sample country.

To encapsulate the state of public health, I use four commonly adopted indicators in the literature as proxies for population health: life expectancy at birth, the crude death rates per 1,000 people,^3^ child mortality rates under five per 1,000 live births, and suicide mortality rates per 100,000 people.

In the preceding theoretical analysis, the ways in which economic uncertainty impacts public health are divided into direct and indirect channels. The indirect channels-economic growth, healthcare expenditure, and environmental pollution-are relatively straightforward to quantify. However, the direct channel, which pertains to psychological disorders with typical symptoms of depression, psychopathy, and aggressiveness, is more challenging to measure due to the lack of available data at the country level for the sample countries. While it is not always the case that suicides are the result of psychological disorders (51, 52), a significant proportion of suicides are indeed closely linked to such conditions (53). Therefore, I use suicide mortality rates as a rough indicator of the nationwide prevalence of psychological disorders to examine the direct effects of economic uncertainty on health.

Consistent with previous studies, economic growth is represented by GDP *per capita* in 2017 international dollars, while healthcare expenditure is gauged by healthcare expenditure *per capita* in 2017 international dollars. Following Neves et al. (54), Munir (55), and Ongan et al. (56), environmental pollution is measured by CO_2_ emissions in metric tons *per capita*. Health indicators, healthcare expenditure, and carbon emissions were sourced from the World Bank database, with GDP data from the IMF WEO database. Barring the economic uncertainty proxy, all variables are taken as logarithms.^4^

### 4.2. Results of baseline models

#### 4.2.1. Model estimation and convergence diagnosis

To optimize computational efficiency and maintain a parsimonious model specification, only one health indicator is included in each model. This strategy results in five panel VAR models, each employing different health proxies.^5^ Following the common practice for annual panel data and guided by the results explicated in Table A2 in Appendix A,^6^ I set the VAR lag length to 1 in the baseline models. The models are estimated by an MCMC sampling procedure with 55,000 replications, of which the initial 45,000 replications are used for burn-in, retaining 10,000 effective draws.^7^

To confirm the convergence of the posterior draws, inefficiency factors for VAR coefficients and covariance are computed. In the model incorporating life expectancy,^8^ the inefficiency factors for the VAR coefficients and covariance fall within relatively low ranges of (0.7298, 1.3304) and (1.08, 1.389), respectively. These results signify the excellent performance of the MCMC draws, rendering them suitable for further inference.

#### 4.2.2. Responses of the macroeconomy to economic uncertainty shocks

I subsequently direct the analysis towards the impulse response functions (IRFs) estimated using posterior MCMC draws to how economic uncertainty impacts population health. Figure B1A in Appendix B illustrates the macroeconomic dynamics following a 1% surge in economic uncertainty. To concentrate on the consequences of the shock, I refrain from displaying the response of the shock variable itself. As depicted in the figure, an upsurge in economic uncertainty precipitates significant reductions in economic growth, environmental pollution, and healthcare expenditure.

The marked response of real GDP to the uncertainty shocks is consistent with the majority of uncertainty literature, including Bachmann et al. (57), Baker et al. (4), and Baker et al. (58). These studies collectively highlight the detrimental impact of economic uncertainty on economic output. Moreover, the response of environmental pollution aligns, to some extent, with the findings by Ahmed et al. (59). However, recent evidence regarding the impact of economic uncertainty on environmental pollution remains contentious. While Adams et al. (60), Atsu and Adams (61), Adedoyin et al. (62), and Yu et al. (63) claim that heightened economic uncertainty causes increased carbon emissions, Abbasi and Adedoyin (64) and Zhang et al. (65) find no discernible impact of EPU on carbon emissions. Furthermore, it is worth considering the adverse impact of economic uncertainty on healthcare expenditure, as it presents a contrasting viewpoint to the findings of (66) who observe positive effects of EPU on healthcare expenditure in China. However, this partially aligns with the conclusions drawn by Cheng and Witvorapong (67), who argue that healthcare policy uncertainty diminishes healthcare expenditure in the United States.

The above findings raise the question: why do environmental pollution and health expenditure respond negatively to economic uncertainty? To answer this question, I estimate the IRFs of shocks to GDP and CO_2_ emissions, as shown in Figure B1B in Appendix B. An increase in economic growth is likely to cause more severe environmental pollution and higher healthcare expenditures. As the economy grows, social investments and consumption increase in tandem, leading to higher environmental pollution, consistent with Davis (68), who states that economic downturns often coincide with improvements in the natural environment. This, in turn, necessitates greater healthcare expenditure to manage the deteriorating environment (as depicted in the right panel of Figure B1B). An increment in GDP also raises national income, endowing the nation with improved abilities to fund healthcare. These findings support the well-known Environmental Kuznets curve hypothesis, which presumes that higher economic output correlates with reduced environmental quality in EMDCs, see Tucker (69), Grossman and Krueger (70), and Zhang and Zhang (71), among others. Moreover, I demonstrate the synchronicity of health expenditure with economic growth and environmental pollution, consistent with the recent study by Ampon-Wireko et al. (72), who propose that carbon emissions and income growth are significant factors influencing healthcare expenditure in non-OCED countries. Thus, following a hike in economic uncertainty, economic growth declines, and environmental pollution and healthcare expenditure tend to decrease correspondingly.

#### 4.2.3. Responses of health indicators to economic uncertainty shocks

**Figure 1:**
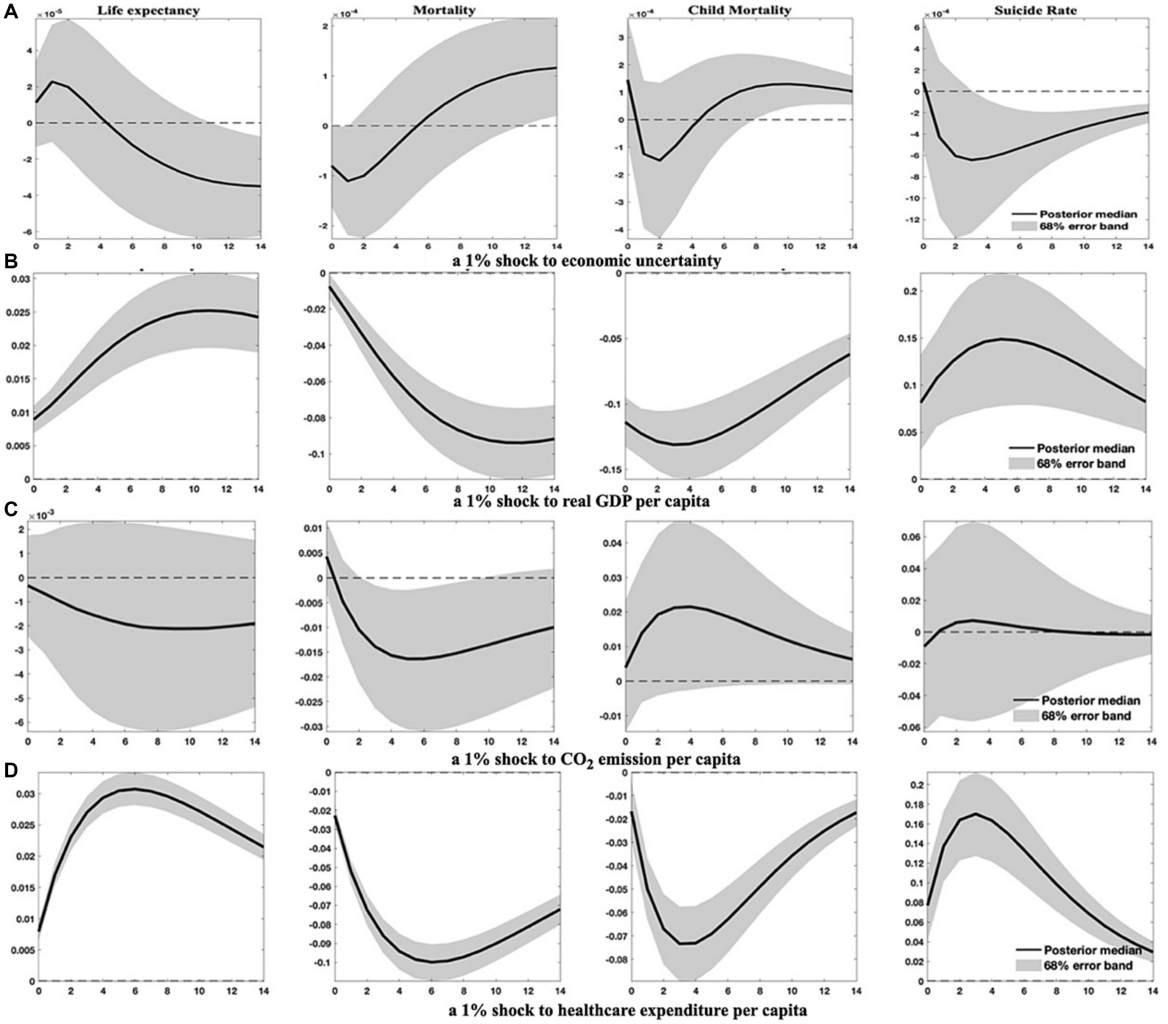
Health indicator dynamics in response to specified shocks. This figure elucidates how health indicators, such as life expectancy, aggregate mortality, child mortality, and suicide rates, react to 1% increases in economic uncertainty, economic growth, environmental pollution, and healthcare expenditure. Solid lines represent the median of posterior estimates of the impulse response functions (IRFs). The area shaded in grey is the 68% error band, constructed from the 16th and 84th percentiles of posterior estimates of the IRFs.

Similarly, the IRFs of health indicators to a 1% shock to economic uncertainty are computed and displayed in Figure 1. The first health indicator, life expectancy, as shown in Figure 1A, undergoes a marginal and statistically insignificant increase in the 2 years following an upsurge in economic uncertainty. However, the median IRF of life expectancy progressively decreases thereafter, turning negative response approximately 5 years after the shock and converging around the ninth year. Moreover, as reported in Figure 1C, an increase in environmental pollution is followed by a persistent decline in life expectancy. However, the corresponding error band is too wide to encompass zero, suggesting environmental pollution may deteriorate population health. This finding is consistent with Steinberger et al. (73), who content that life expectancy weakly correlates with carbon emissions. Brunner and Maruyama (74), conversely, report a positive association between life expectancy and CO_2_ emissions in low-income and lower-middle-income countries. Given the reactions of environmental pollution to economic uncertainty and economic growth (as shown in Figure 1B), the initial health gains post-shock may be attributed to the environmental improvements resulting from economic downturns. Nevertheless, as disclosed in Figures 1B,D, buildups in output and healthcare expenditure are critical drivers of life expectancy improvement (75, 76). Hence, the health detriments from contractions in economic growth and healthcare expenditure become apparent in the long run, evincing a significant and persistent diminution in life expectancy.

The remainder of Figure 1 illustrates the effects of distinct shocks on aggregate mortality (crude death rates), child mortality, and suicide rates. The IRFs depicted in Figures 1B–D compellingly demonstrate that augmentations in economic growth and healthcare spending significantly contribute to the reduction of both aggregate mortality and child mortality in EMDCs, corroborating the evidence reported in Nishiyama (77). Conversely, an increase in environmental pollution may engender a subtle and persistent rise in child mortality across the response horizon, alongside a marginal leap in aggregate mortality within a year, affirming the findings of Bressler (78).

However, as Figure 1 conveys, the response of aggregate mortality shifts from a negative trajectory in the first 6 years after a 1% uncertainty shock to a positive one thereafter. This suggests that while an increase in economic uncertainty may initially reduce aggregate mortality in the short term due to improved environmental quality, it could considerably impair public health by raising mortality rates in the wake of economic downturns. This inference diverges from the conclusion drawn by Ahmad et al. (40), who posit that CO_2_ emissions have no significant role in adult mortality in developing countries. In contrast, the response of child mortality to uncertainty shocks oscillates dramatically between positive and negative trajectories. Specifically, child mortality rises following uncertainty shocks but swiftly descends into the negative region for approximately 3 years before reverting and converging to a significant level. This indicates that the economic growth channel may dominate and substantially increase child mortality in the long run.

Pertaining to suicide rates, they exhibit nominal responses to environmental pollution shocks but react significantly and positively to economic growth and healthcare expenditure shocks. This observation substantiates Lynn’s hypothesis (79, 80) that higher economic growth can lead to anxiety and elevate suicide rates. Additionally, heightened economic uncertainty initially triggers a minor increase in suicide rates, providing additional evidence to support the findings of Vandoros et al. (10) and Claveria (13). In the long run, nevertheless, rises in economic uncertainty, alongside declines in economic output and healthcare expenditure, can significantly lower suicide rates.

### 4.3. Robustness checks

To assess the sensitivity of the results from the baseline models, I consider two alternative model specifications: (1) establishing a larger VAR lag length, and (2) incorporating additional relevant variables.

**Figure 2:**
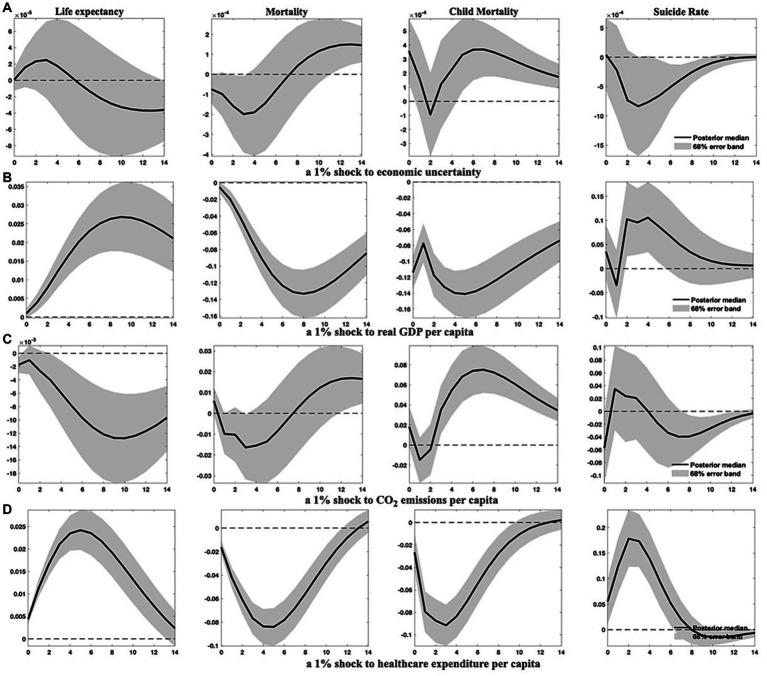
Health indicator responses to specified shocks in panel VAR (3) models. This figure presents how four health indicators evolve following 1% increases in economic uncertainty, economic growth, environmental pollution, and healthcare expenditure, when the VAR lag length is set at 3. Solid lines denote the median of posterior estimates of the IRFs, while the grey-shaded area stands for the 68% error band constructed using the 16th and 84th percentiles of posterior estimates of the IRFs.

First, I set a larger VAR lag length of 3, capturing almost a complete Kitchin cycle, and re-estimate the models. The estimated IRFs under this specification are presented in Figure 2; Figure B2 in Appendix B.^9^

Comparatively and generally, setting a larger VAR lag length does not substantially alter the results from the baseline models. However, the impact of environmental pollution on health indicators becomes more conspicuous, suggesting that environmental degradation has significantly delayed adverse effects on public health.

**Figure 3:**
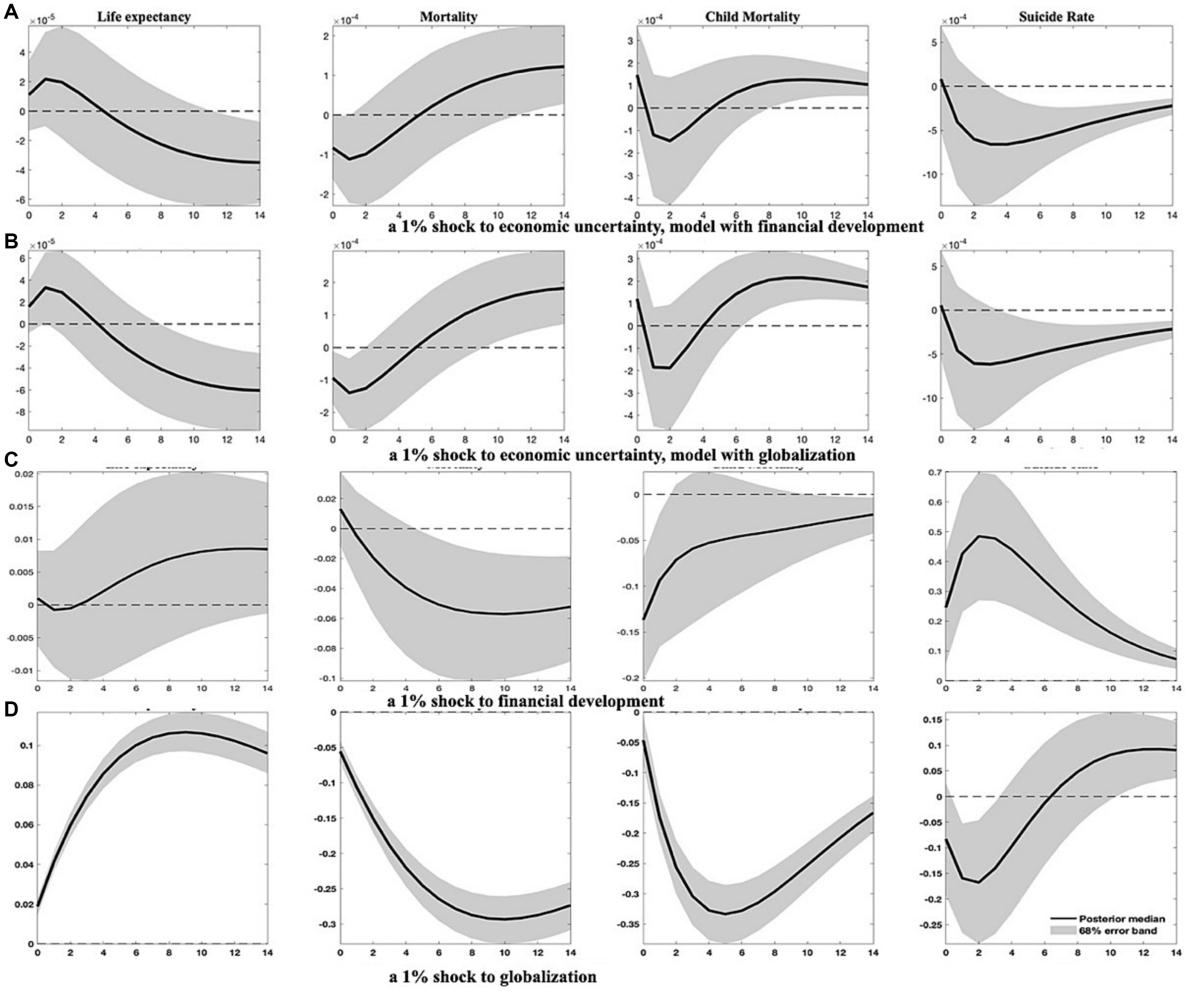
Health indicator responses to specified shocks in augmented models. This figure outlines how four health indicators respond to increases in economic uncertainty, as well as improvements in financial development and globalization. As in previous figures, solid lines represent the median of posterior estimates of the IRFs, and the grey-shaded regions denote the 68% error band constructed from the 16th and 84th percentiles of posterior estimates of the IRFs.

Second, I integrate an additional variable into the baseline models to verify the robustness of the findings. Given the relevance of financial development [proxied by a broad index established by Svirydzenka (81)] and globalization [measured by the KOF index of overall globalization developed by Dreher (82)] to the macroeconomy and their influence on population health, I choose to include these variables individually,^10^ yielding several 6-variate panel VAR(1) models. The IRFs estimated for these augmented models are exhibited in Figure 3; Figure B3 in Appendix B.

As depicted in Figure 3, the impact of economic uncertainty on population health remains substantial. Both models predict less pronounced increases in life expectancy within approximately 2 years following a surge in economic uncertainty, accompanied by a marked decline in aggregate mortality. Child mortality and suicide rates exhibit less responsiveness to economic uncertainty shocks in the short run. However, a discernible rise in child mortality and a decline in suicide rates transpire about 5 years after an upswing in economic uncertainty.

In addition, the mechanism through which economic uncertainty impacts population health could be accentuated by financial development and globalization. As shown in Figures B3B,C, EMDCs generally benefit from invigorated globalization and financial development, which stimulate economic growth and improve population health. Precisely, heightened globalization unequivocally yields positive outcomes for all health indicators, corroborating the findings of Cole and Neumayer (83). Upgraded financial development significantly reduces child mortality and has a lagged effect in reducing aggregate mortality. However, advancements in financial development may not be conducive to lower suicide rates. Notwithstanding the favorable implications of financial development and globalization, elevated economic uncertainty impedes these two variables, as shown in Figure B3A.

### 4.4. Heterogeneity analysis

**Figure 4:**
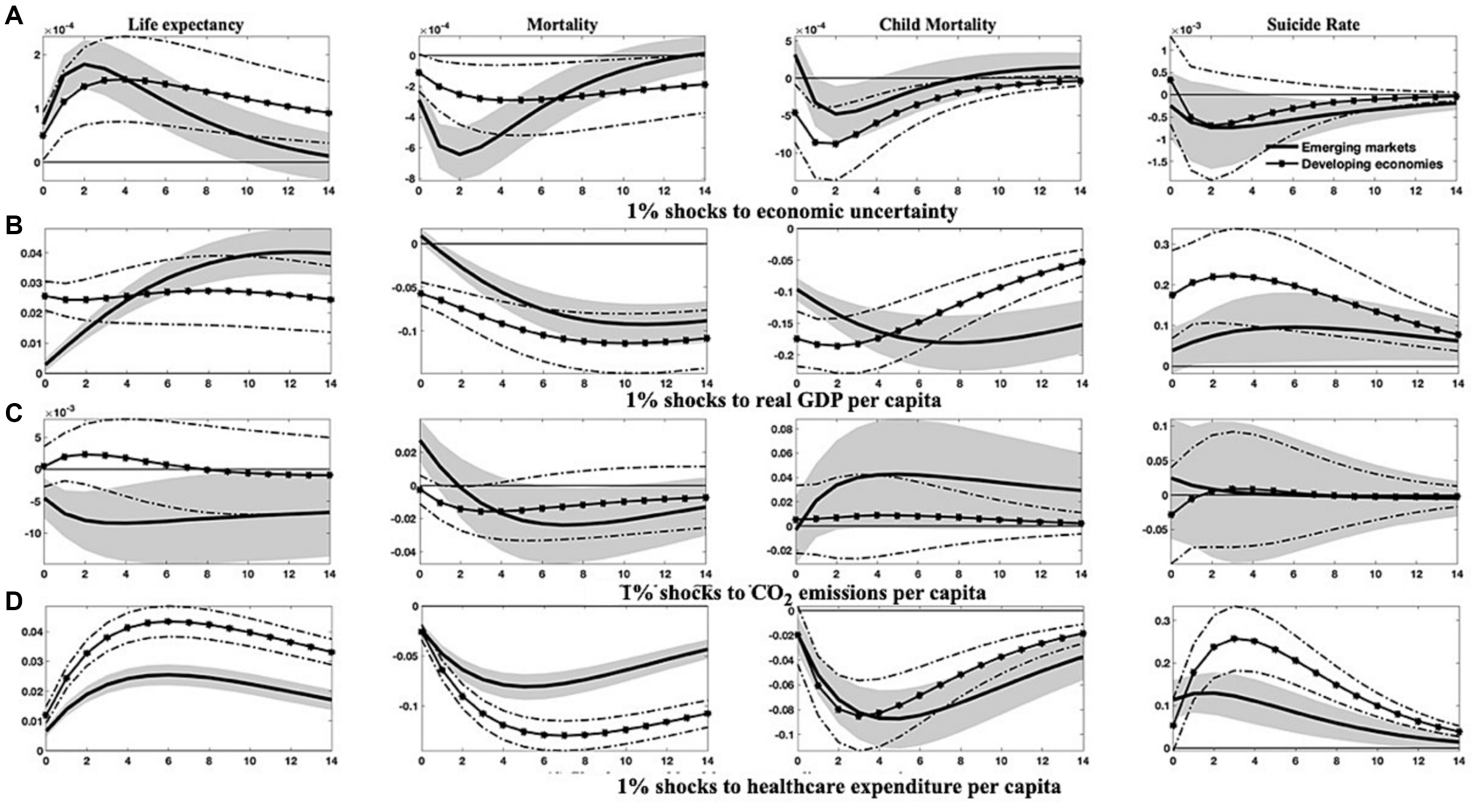
Differential responses of health indicators to 1% shocks to specific variables in emerging markets and developing countries. This figure differentiates the changes in four health indicators between emerging markets and developing countries in reaction to 1% increases in economic uncertainty, economic growth, environmental pollution, and healthcare expenditure. Solid lines and solid asterisk-marked lines denote the median of the posterior estimates of the corresponding IRFs, while grey-shaded regions and dash-dotted lines represent the 68% error bands constructed from the 16th and 84th percentiles of the posterior estimates of the corresponding IRFs.

**Figure 5:**
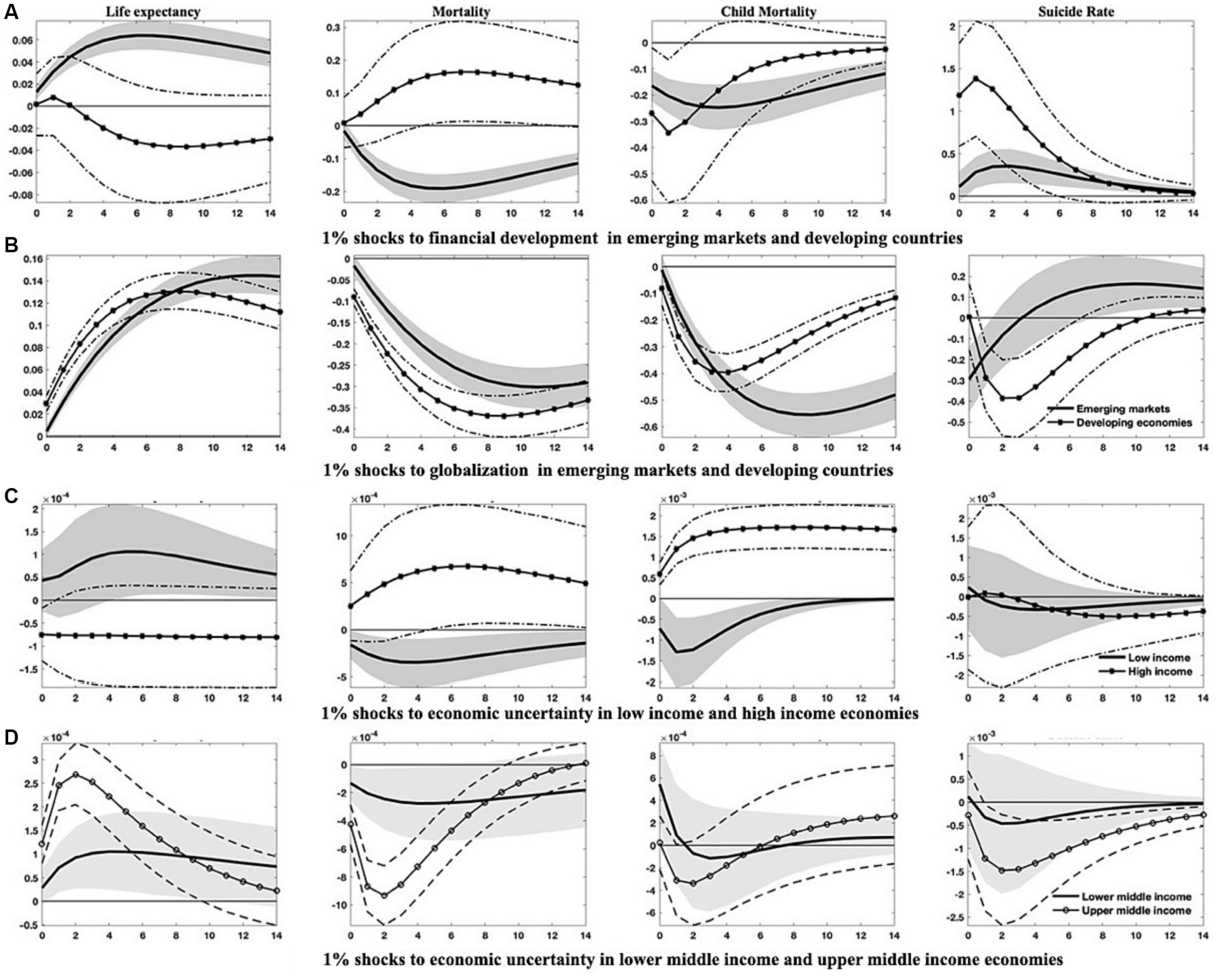
Responses of health indicators to 1% shocks in financial development, globalization, and economic uncertainty across varied countries. This figure delineates (1) the differential trajectories of health indicators between emerging markets and developing countries following enhancements in financial development and globalization, and (2) the varied responses of health indicators to surges in economic uncertainty across countries with disparate income levels. Solid lines and solid dot/asterisk-marked lines represent the median of the posterior estimates of the corresponding IRFs, while grey-shaded regions and dash(-dotted) lines are the 68% error bands constructed using the 16th and 84th percentiles of the posterior estimates of the corresponding IRFs.

**Figure 6:**
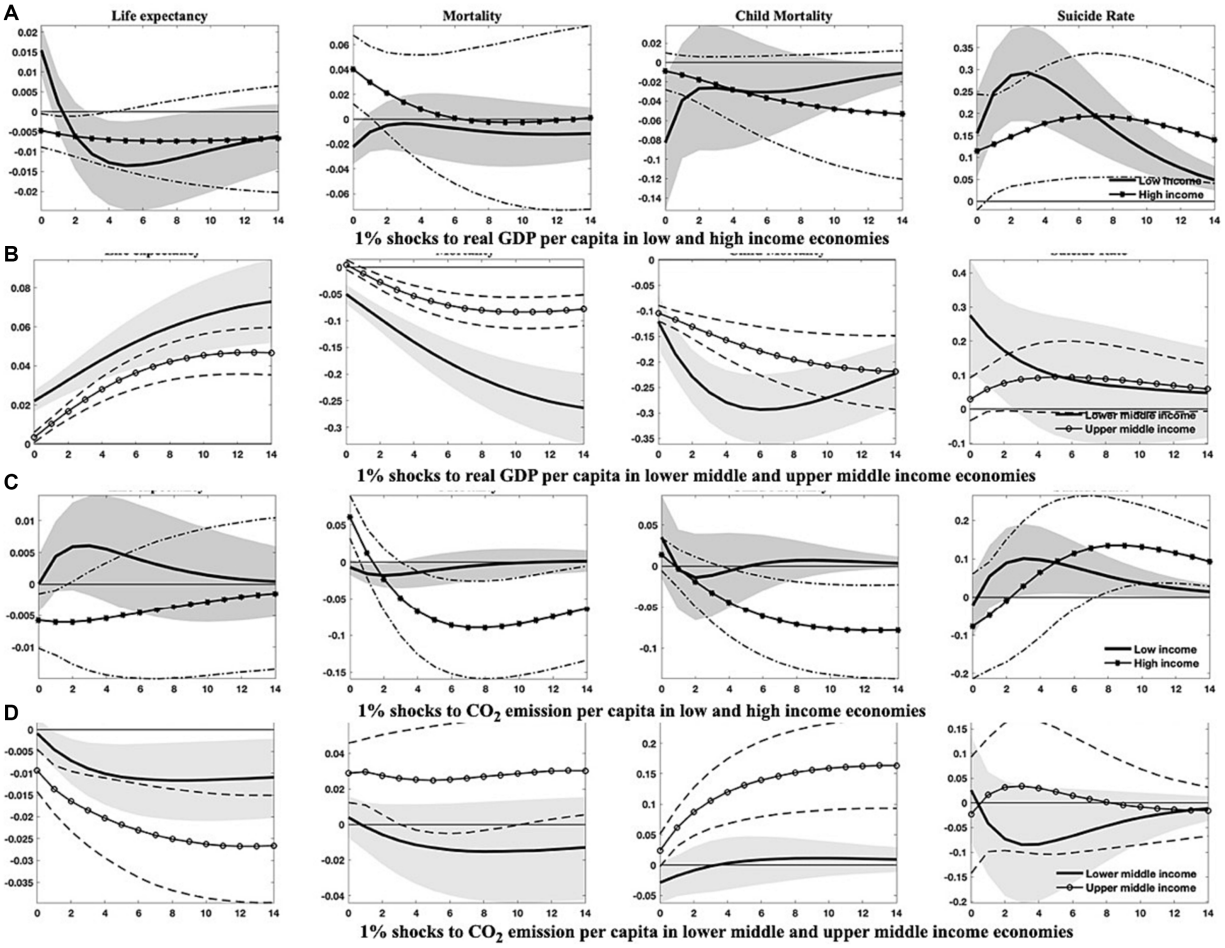
Responses of health indicators to 1% shocks to real GDP *per capita* and CO_2_ emissions across economies with diverse income levels. This figure outlines the distinct dynamics of health indicators in response to economic growth and environmental pollution among countries of varying income levels. Soild lines and solid dot/asterisk-marked lines symbolize the median of posterior estimates of the corresponding IRFs, while grey regions and dash(-dotted) lines indicate the 68% error bands derived from the 16th and 84th percentiles of posterior estimates of the corresponding IRFs.

**Figure 7:**
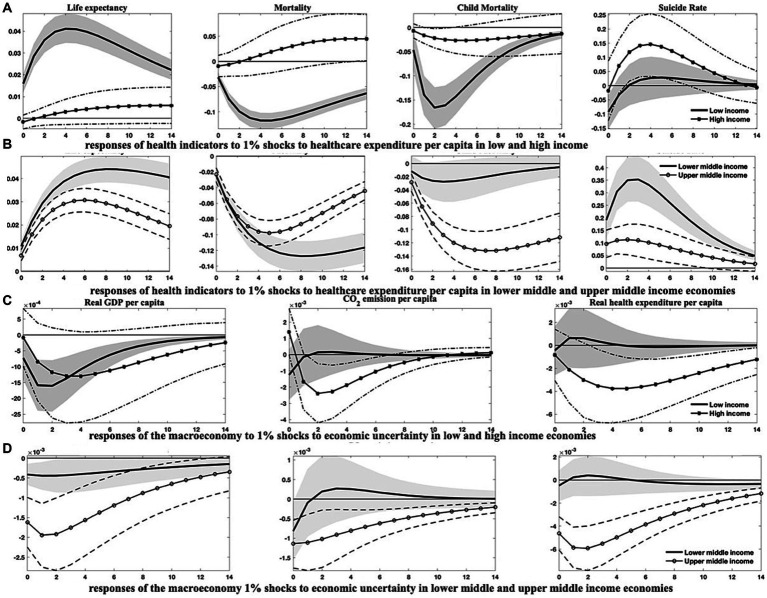
Responses of health indicators to 1% shocks to healthcare expenditure *per capita* and macroeconomic responses to 1% shocks to economic uncertainty across economies of diverse income levels. This figure demonstrates (1) the varied dynamics of health indicators in response to growth in healthcare expenditure among countries with differing income levels, and (2) the differential responses in economic growth, environmental pollution, and healthcare expenditure in the face of escalating economic uncertainty across countries of disparate income levels. Soild lines and solid dot/asterisk-marked lines depict the median of posterior estimates of the corresponding IRFs, while grey regions and dash(-dotted) lines indicate the 68% error bands constructed from the 16th and 84th percentiles of posterior estimates of the corresponding IRFs.

The findings from the above analysis indicate that economies with higher income levels frequently exhibit better population health, owing to their typically greater healthcare expenditure, advanced financial development, and a higher degree of globalization. Higher-income levels are associated with increased demand for environmental protections (69), promoting governments to pursue eco-friendly economic growth strategies that ultimately enhance overall health conditions. As a result, heterogeneities may emerge due to disparities in development. To investigate this proposition, I divide the sample into (1) emerging markets and developing economies, as well as (2) countries categorized by four income tiers-low, lower-middle, upper-middle, and high-income levels. I then re-estimate the models to compile the relevant empirical results presented in Figures 4–7; Figures B4–B6 in Appendix B.^11^

#### 4.4.1. Comparison between emerging markets and developing countries

The IRFs depicted in Figures 4, 5; Figure B4 provide compelling evidence for the heterogeneous effects across these distinct country classifications. Specifically, after an upsurge in economic uncertainty, both emerging markets and developing countries exhibit discernible improvements in population health. The responses of life expectancy and mortality rates in emerging markets, along with the child mortality rates in developing countries, appear more significant. However, economic uncertainty seems to have minimal impact on suicide rates within both country classifications, particularly in the short term. Excepting suicide rates, the health gains generated from economic growth, healthcare expenditure, and globalization are appreciably more substantial in developing countries than in emerging markets.

Contrastingly, health indicators for both country classifications respond markedly and differently to shocks in environmental pollution and financial development. It is noteworthy that the adverse effects of environmental pollution and the salutary effects of financial development on life expectancy and mortality rates are more evident in emerging markets, whereas developing countries demonstrate lesser responsiveness to these shocks.

In the aftermath of an escalation in economic uncertainty, emerging markets suffer more considerable drops in economic output, environmental pollution, healthcare expenditure, and financial development. In comparison, developing countries confront more drastic reductions in the degree of globalization.

#### 4.4.2. Comparison among countries with different income levels

The differences among countries at varying income levels are starkly apparent in this study. Regarding health outcomes resulting from economic uncertainty (Figures 5C,D), all economies, except those at high-income levels, are likely to see significant improvements in public health after a hike in economic uncertainty. Conversely, high-income countries may withstand an increase in aggregate mortality and child mortality rates, as well as a transient diminution in life expectancy. Among all income categories, the responses of health indicators to uncertainty shocks are the most pronounced in upper-middle-income countries.

Concerning other health-influencing factors, it is evident that upper-middle-income and notably lower-middle-income countries could realize substantial benefits from progressive economic growth and enhanced healthcare expenditure, like the potential gains for low-income countries with enlarged healthcare expenditure. These advantages are observable in the prolonged life expectancy and reduced mortality rates (Figures 6, 7). However, it should be noted that in low-income countries, health improvements attributable to economic growth tend to be relatively modest and less persistent, as shown in Figure 6A. In contrast, health indicators in high-income countries demonstrate a lower sensitivity to fluctuations in economic growth and healthcare expenditure, with the notable exception of suicide rates, which may comove positively with economic growth and healthcare expenditure across all country classifications. In the context of mounting environmental pollution (Figures 6C,D), upper-middle-income countries are likely to confront significant health deterioration, manifested by curtailed life expectancy and increased mortality rates, including both aggregate and child mortality. Conversely, high-income countries may only endure a slight decrement in life expectancy, along with an increment in aggregate mortality in the short run. Yet, health indicators in other income groupings exhibit negligible responses to changes in environmental pollution.

Furthermore, as illustrated in Figures 7C,D, economic growth, environmental pollution, and healthcare expenditure in countries of varying income levels display distinct dynamics after a rising shock in economic uncertainty. Following an uptick in economic uncertainty, low-income and upper-middle-income countries would experience more stringent economic contractions than lower-middle-income countries, while high-income countries show a reduced reactivity. Additionally, concerning environmental pollution and healthcare expenditure, shocks from economic uncertainty exert an immediate and persistent impact on upper-middle-income countries, with a delayed effect on high-income countries.

Upon considering the income levels of different countries, the favorable health outcomes associated with financial development do not consistently manifest across all nations. The IRFs in Figures B5A,B in Appendix B intimate that the health benefits of financial development are predominantly evident in upper-middle-income countries. Paradoxically, financial development could potentially precipitate adverse effects on health indicators within high-income countries. Globalization, meanwhile, ostensibly fosters positive health effects, particularly within low-income, lower-middle-income, and upper-middle-income countries (Figures B5C,D). For low-income and upper-middle-income countries, the benefits conferred by globalization may be negated by economic uncertainty, which has been identified as a significant factor in attenuating globalization degrees within these countries (Figure B6 in Appendix B). Interestingly, lower-middle-income countries could potentially glean health gains from economic uncertainty, given that it tends to impose impediments to financial development in both lower-middle-income and high-income countries (Figure B6 in Appendix B).

## 5. Conclusion

In recent years, the global economy has been marked by unprecedented levels of uncertainty. This has sparked extensive academic interest, particularly concerning the subsequent effects on economic activities, agent behavior, and asset prices. Theoretically, economic uncertainty impacts public health through a direct psychological channel and three indirect channels: economic growth, healthcare expenditure, and environmental quality. This paper turns its focus to emerging markets and developing countries (EMDCs), as these regions are most vulnerable to increased economic uncertainty due to their inadequate medical infrastructure and services. I assess the impact of economic uncertainty on public health by analyzing a comprehensive annual, country-level dataset for 103 countries through a Bayesian panel VAR model developed by Beetsma et al. (20).

Based on the entire sample, the empirical findings reveal a transient and marginal beneficial health impact from heightened economic uncertainty. This is evidenced by increased life expectancy and reduced mortality rates. However, in the long run, nations tend to experience an overall decline in health due to persistent decreases in economic growth and healthcare expenditure, both of which are crucial determinants of health. Robustness checks confirm that the results remain largely unaffected by VAR lag length and model dimensionality. Additionally, environmental pollution exerts significant detrimental effects on health. I also present further evidence supporting the positive health outcomes associated with financial development and globalization, both of which respond negatively to economic uncertainty shocks.

The subsample analysis underscores considerable heterogeneities across diverse types of countries within EMDCs. I concentrate on three health indicators—life expectancy, aggregate mortality, and child mortality—and find that the health-enhancing effects of economic uncertainty are more pronounced in emerging markets, low-income, and upper-middle-income countries. Notably, surges in economic uncertainty may trigger increased child mortality rates in high-income and lower-middle-income nations. Additionally, the health benefits derived from healthcare expenditure are more apparent in developing, low-income, and lower-middle-income countries, while economic growth may not consistently improve health outcomes in high-income countries. The deleterious effects of environmental pollution on health are predominantly observable in emerging markets, lower-middle-income, and upper-middle-income countries. Lastly, financial development does not significantly affect health outcomes for developing and high-income countries. In contrast, globalization has a positive health impact for all classifications of nations except high-income countries. Following escalations in economic uncertainty, only upper-middle-income countries exhibit a substantial decline in suicide rates. Contrary to common belief, economic growth and healthcare expenditure may not contribute to reduced suicide rates in countries other than low-income nations. Furthermore, I explore the interlinkages among economic uncertainty, environmental pollution, and healthcare expenditures, within the context of this study, themes which have been extensively examined in previous studies.

The findings yield several policy implications. First, EMDCs should be aware of the potentially detrimental impact of economic uncertainty and contemplate implementing proactive measures to mitigate the adverse effects of economic downturns during periods of heightened economic uncertainty, such as social safety nets and targeted healthcare interventions. Second, EMDCs should continue investing in their healthcare systems, as they have been demonstrated to be salient factors contributing to positive health outcomes (84). Third, EMDCs need to be vigilant of potential health detriments resulting from environmental degradation and endeavor to pursue eco-friendly economic growth trajectories. Fourth, EMDCs should strive to develop a more sophisticated financial system and enhance globalization, promoting economic development and public health. Lastly, EMDCs should consider enacting policies that reduce economic uncertainty, such as refining the business environment, augmenting the credibility and transparency of economic policies, and encouraging economic diversification. These actions may facilitate sustainable economic growth and, consequently, improve population health.

Moreover, the findings provide critical insights into the primary policy interventions for enhancing public health in countries of varying income levels. For low-income countries, it is essential to foster economic growth, bolster globalization, and make considerable investments in healthcare expenditure, particularly in times of significant economic uncertainty (85). Lower-middle-income countries should prioritize interventions to reduce child mortality rates that may increase amid economic uncertainty, while also endeavoring to embrace globalization to reap health benefits. In the case of upper-middle-income countries, the expansion of healthcare access and coverage during economic uncertainty is recommended. Additionally, these countries should strengthen environmental regulations to improve environmental quality and promote financial inclusion to maximize the positive effects of financial development (86). High-income economies, on the other hand, should implement policies to mitigate the adverse health impacts of economic uncertainty, with a particular emphasis on child mortality. Investment in green technologies and renewable energy is necessary to address environmental degradation and its detrimental health effects (87, 88). In addition, these economies should strive to optimize the efficiency of healthcare expenditure, monitor suicide rates diligently, and ensure the provision of adequate mental health resources during periods of heightened economic uncertainty.

Notwithstanding, this study retains several limitations that warrant further investigation. For example, the increasing integration of economies and financial markets worldwide necessitates accounting for international spillovers and dynamic interdependencies between countries (89, 90). Nonetheless, the sample used in this study precludes extensive examination of cross-country interdependencies. A potential future study could concentrate on a specific region with a limited number of countries and employ the panel VAR model with the stochastic search specification selection algorithm proposed by Koop and Korobilis (91) to allow for all potential dynamic interdependencies and heterogeneities. In addition, regime shifts might prevail in the relationship between health and its determinants due to economic and political events. Future studies could employ models incorporating potential regime shifts, such as Markov-regime switching and time-varying parameters models, to offer more insights. Furthermore, the extant literature has uncovered many other economic and social variables influencing health, such as income inequality, democracy, military expenditure, natural resource dependence, and education. However, I primarily excluded these variables from this study due to data availability constraints and the desire for model parsimony. Incorporating these variables to construct a more comprehensive model is left for future work.

## Data availability statement

The original contributions presented in the study are included in the article/Supplementary Material, further inquiries can be directed to the corresponding author.

## Author contributions

LL: Writing – original draft, Writing – review & editing.

## Footnotes

^1^Here, I briefly discuss the principles and routines of the algorithm; for further implementation and theories about the algorithm, the reader is referred to Bayesian econometrics textbooks.

^2^The outbreak of the COVID-19 pandemic in 2020 propelled world economic uncertainty to unprecedented levels and imposed tremendous threats to public health. Including data from 2020 onward in the analysis may introduce a bias that health and economic uncertainty are highly correlated. Therefore, considering this backdrop and data availability, I limit the sample to 2019.

^3^In the following analysis, I will refer to mortality or aggregate mortality.

^4^The WUI occasionally contains zeros, indicating that the absence of uncertainty-related words in the report. Taking the logarithm in such cases can lead to errors. If I apply logarithmic transformation to the WUI while retaining the zeros, it may significantly diminish the fluctuations of the WUI, potentially producing biased results.

^5^One mild caveat is that this strategy overlooks potential intercorrelations among health indicators. However, specifying a model that accommodates all possible intercorrelations presents a conundrum. Incorporating all health indicators into a single model could increase dimensionality and, consequently, the computational complexity, even though it might improve model fitness.

^6^In Bayesian econometrics, model performance evaluation often hinges on the deviance information criterion (DIC). Nevertheless, drawing inferences from this statistic is a formidable challenge given the large country-level units. Thus, despite their intrinsic differences from the Bayesian approach, I have chosen to rely on information criteria statistics derived from frequentist estimation methods.

^7^I did not perform panel unit root tests to validate variable stationarity. This is because such a condition is not required in panel VAR model settings or Bayesian econometrics.

^8^Models with other health indicators generate similar results, which are available upon request.

^9^In this section, I will not report the IRFs like those in Figure 1B, as they are not my primary focus. However, these IRFs are available upon request.

^10^Following the literature, financial development and globalization are ordered before economic output.

^11^My emphasis in this section is primarily on the baseline models. However, to provide further insights into the importance of financial development and globalization, I also examine augmented models that incorporate these two variables.
