# Supplementary material for: Economic uncertainty and population health: insights from emerging markets and developing countries
Source: Front Public Health. 2023 Oct 25;11:1292236. doi: 10.3389/fpubh.2023.1292236 (PMC10634310; doi:10.3389/fpubh.2023.1292236)
Supplement: Supplementary file 1 [file Data_Sheet_1.docx]

**Supplementary Material for “Economic Uncertainty and Population Health: Insights from Emerging Markets and Developing Countries”**

**Appendix: Tables and Figures**

**A: Tables**

**Table A1:** Sample countries and classification

| **Africa** (49) |
| --- |
| Angola(EM,LMI), United Arab Emirates(EM,HI), Burundi(DC,LI), Benin(DC,LMI), Burkina Faso(DC,LI), Botswana(EM,UMI), Central African Republic(DC,LI), Côte d'Ivoire(DC,LMI), Cameroon(DC,LMI), Democratic Republic of the Congo(DC,LI), Republic of Congo(DC,LMI), Algeria(EM,LMI), Egypt(EM,LMI), Eritrea(DC,LI), Ethiopia(DC,LI), Gabon(EM,UMI), Ghana(DC,LMI), Guinea(DC,LI), Guinea-Bissau(DC,LI), Islamic Republic of Iran(EM,UMI), Jordan(EM,UMI), Kenya(DC,LMI), Kuwait(EM,HI), Lebanon(EM,UMI), Libya(EM,UMI), Lesotho(DC,LMI), Morocco(EM,LMI), Madagascar(DC,LI), Mali(DC,LI), Mozambique(DC,LI), Mauritania(DC,LMI), Malawi(DC,LI), Namibia(EM,UMI), Niger(DC,LI), Nigeria(DC,LMI), Oman(EM,HI), Qatar(EM,HI), Rwanda(DC,LI), Saudi Arabia(EM,HI), Senegal(DC,LMI), Sierra Leone(DC,LI), Chad(DC,LI), Togo(DC,LI), Tunisia(EM,LMI), Tanzania(DC,LMI), Uganda(DC,LI), Yemen(DC,LI), South Africa(EM,UMI), Zambia(DC,LMI) |
| **East Asia, South Asia, and the Pacific** (15) |
| Bangladesh(DC,LMI), China(EM,UMI), Indonesia(EM,UMI), India(EM,LMI), Cambodia(DC,LMI), Lao P.D.R.(DC,LMI), Sri Lanka(EM,LMI), Mongolia(EM,LMI), Malaysia(EM,UMI), Nepal(DC,LMI), Pakistan(EM,LMI), Philippines(EM,LMI), Papua New Guinea(DC,LMI), Thailand(EM,UMI), Vietnam(EM,LMI) |
| **Europe and Central Asia** (19) |
| Albania(EM,UMI), Armenia(EM,UMI), Azerbaijan(EM,UMI), Bulgaria(EM,UMI), Belarus(EM,UMI), Georgia(EM,UMI), Croatia(EM,HI), Hungary(EM,HI), Kazakhstan(EM,UMI), Kyrgyz Republic(DC,LMI), Moldova(DC,LMI), Poland(EM,HI), Romania(EM,HI), Russia(EM,UMI), Tajikistan(DC,LI), Turkmenistan(EM,UMI), Turkey(EM,UMI), Ukraine(EM,LMI), Uzbekistan(DC,LMI) |
| **Latin America and the Caribbean** (20) |
| Argentina(EM,UMI), Bolivia(EM,LMI), Brazil(EM,UMI), Chile(EM,HI), Colombia(EM,UMI), Costa Rica(EM,UMI), Dominican Republic(EM,UMI), Ecuador(EM,UMI), Guatemala(EM,UMI), Honduras(DC,LMI), Haiti(DC,LI), Jamaica(EM,UMI), Mexico(EM,UMI), Nicaragua(DC,LMI), Panama(EM,HI), Peru(EM,UMI), Paraguay(EM,UMI), El Salvador(EM,LMI), Uruguay(EM,HI), Venezuela(EM,UMI) |

**Notes:** The acronyms EM and DC denote emerging markets and developing countries, respectively. Meanwhile, LI, LMI, UMI, and HI represent low-income, lower-middle-income, upper-middle-income, and high-income countries, respectively. The classifications follow the guidelines of the International Monetary Fund (IMF).

**Table. A2:** Selection of optimal lag length

|  | BIC | AIC | HQIC | BIC | AIC | HQIC |
| --- | --- | --- | --- | --- | --- | --- |
| health indicator: life expectancy | | | | health indicator: mortality | | |
| lag 1 | -57988 | -14869.9 | -32102.8 | -57995 | -14876.9 | -32109.8 |
| lag 2 | -57435 | -14822.4 | -31896 | -57436.7 | -14824.1 | -31897.7 |
| lag 3 | -56470.2 | -14659.6 | -31455.9 | -56471.8 | -14661.2 | -31457.5 |
| lag 4 | -55312.4 | -14453.7 | -30912.9 | -55313.6 | -14454.9 | -30914.1 |
| health indicator: child mortality | | | | health indicator: suicide rates | | |
| lag 1 | -57990.5 | -14872.4 | -32105.3 | -57992 | -14873.9 | -32106.8 |
| lag 2 | -57435.1 | -14822.5 | -31896.1 | -57436.8 | -14824.2 | -31897.9 |
| lag 3 | -56472.3 | -14661.7 | -31458 | -56473.4 | -14662.8 | -31459.1 |
| lag 4 | -56473.4 | -14662.8 | -31459.1 | -55318 | -14459.3 | -30918.5 |

**Notes:** Each model was estimated using the Generalized Method of Moments (GMM) technique. Akaike Information Criterion (AIC), Bayesian Information Criterion (BIC), and Hannan-Quinn Information Criterion (HQIC) were calculated for each model using the methodology proposed by Andrews and Lu (1). The lag length associated with the lowest statistics is preferred.

**B: Figures**


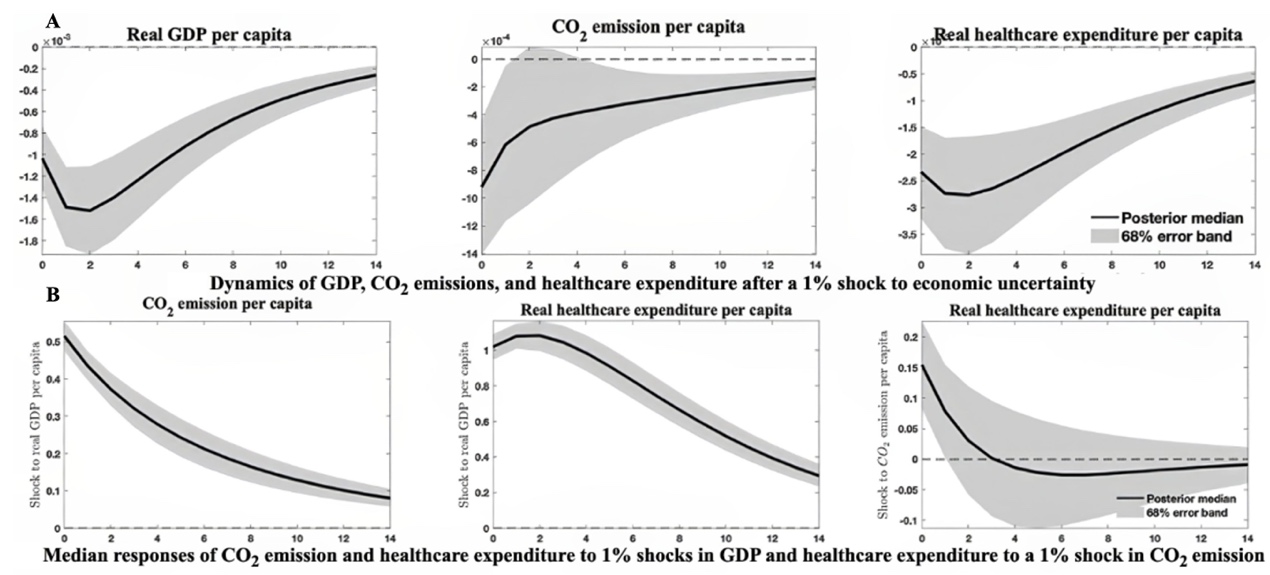


**Figure B1**

Estimated impulse response functions of selected variables. Three key relationships are disclosed in this figure:(1) the consequences of an increase in economic uncertainty on economic growth, environmental pollution, and healthcare expenditure, (2) the influence of economic growth on environmental pollution and healthcare expenditure, and (3) the reaction of healthcare expenditure to changes in environmental pollution. Solid lines represent the median of the posterior estimates of the impulse response functions (IRFs). Grey regions, meanwhile, denote 68% error bands, constructed from the 16th and 84th percentiles of the posterior estimates of the IRFs.


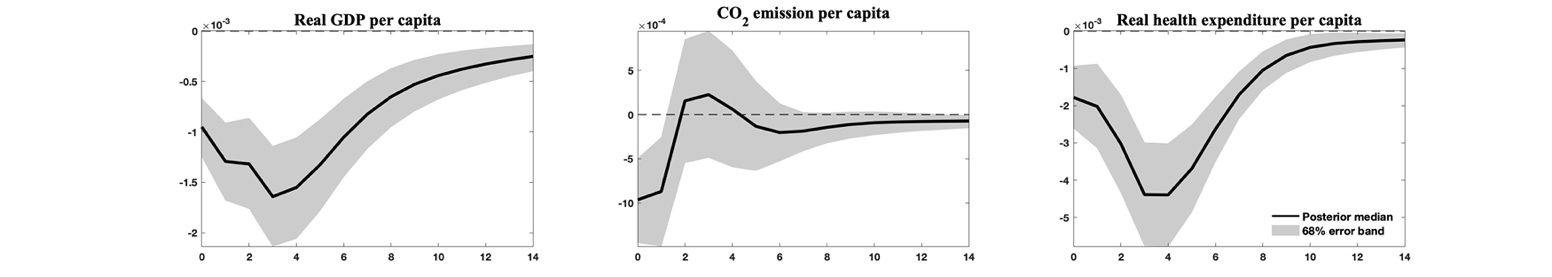


**Figure B2**

Macroeconomic responses to economic uncertainty shocks in panel var(3) models. This figure demonstrates the effects of a rise in economic uncertainty on economic growth, environmental pollution, and healthcare expenditure, precisely when the model's lag length is set to 3. Solid lines represent the median of the posterior estimates of the IRFs, while the grey regions denote 68% error bands constructed from the 16th and 84th percentiles of the posterior estimates of the IRFs.


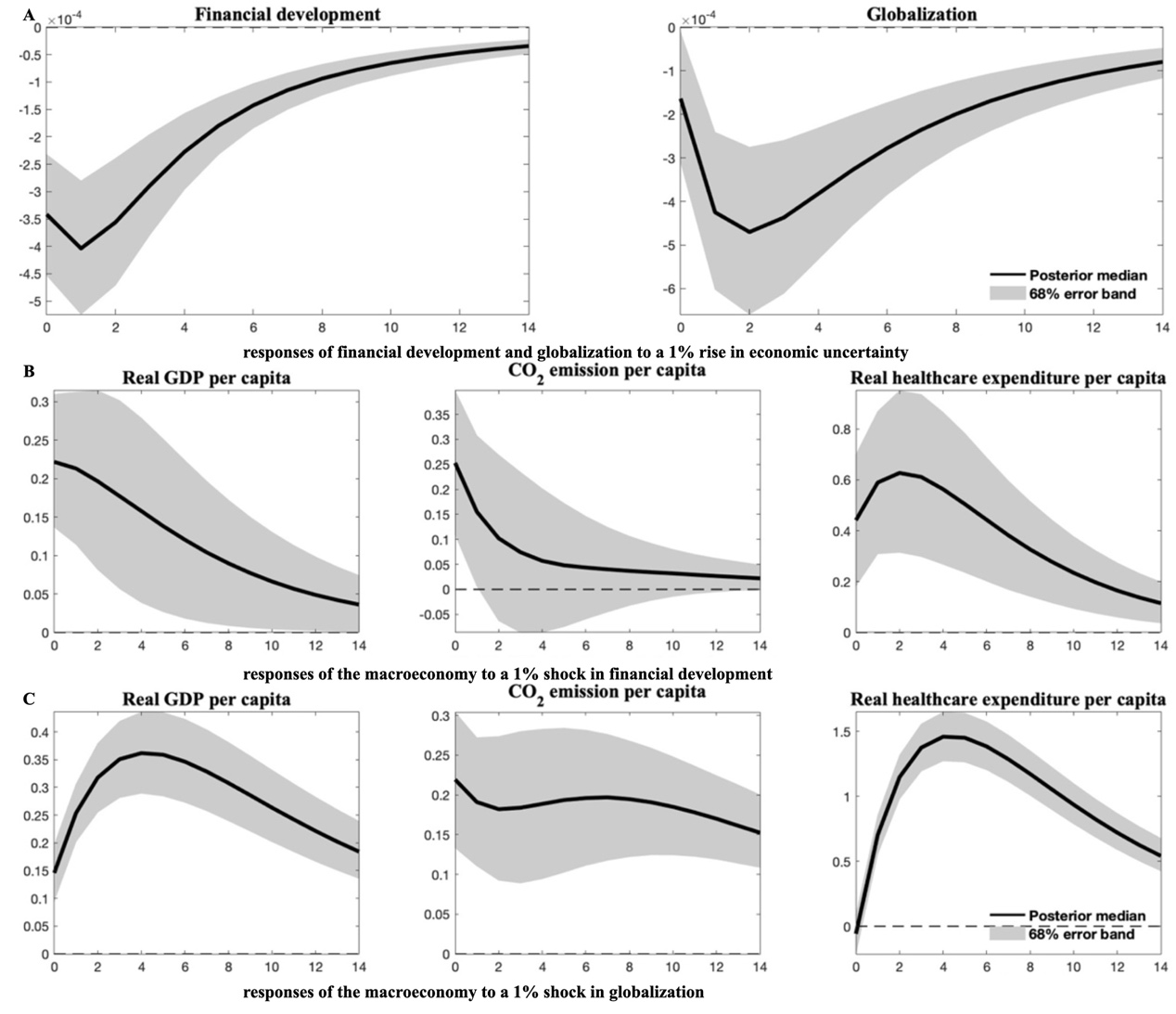


**Figure B3**

Estimated impulse response functions of selected variables in augmented models. This figure conveys two primary relationships: (1) the obstructive impact of increased economic uncertainty on financial development and globalization, and (2) the results of advancements in financial development and globalization on economic growth, environmental pollution, and healthcare expenditure. Solid lines, once again, represent the median of the posterior estimates of the IRFs, while the grey regions denote 68% error bands constructed from the 16th and 84th percentiles of the posterior estimates of the IRFs.


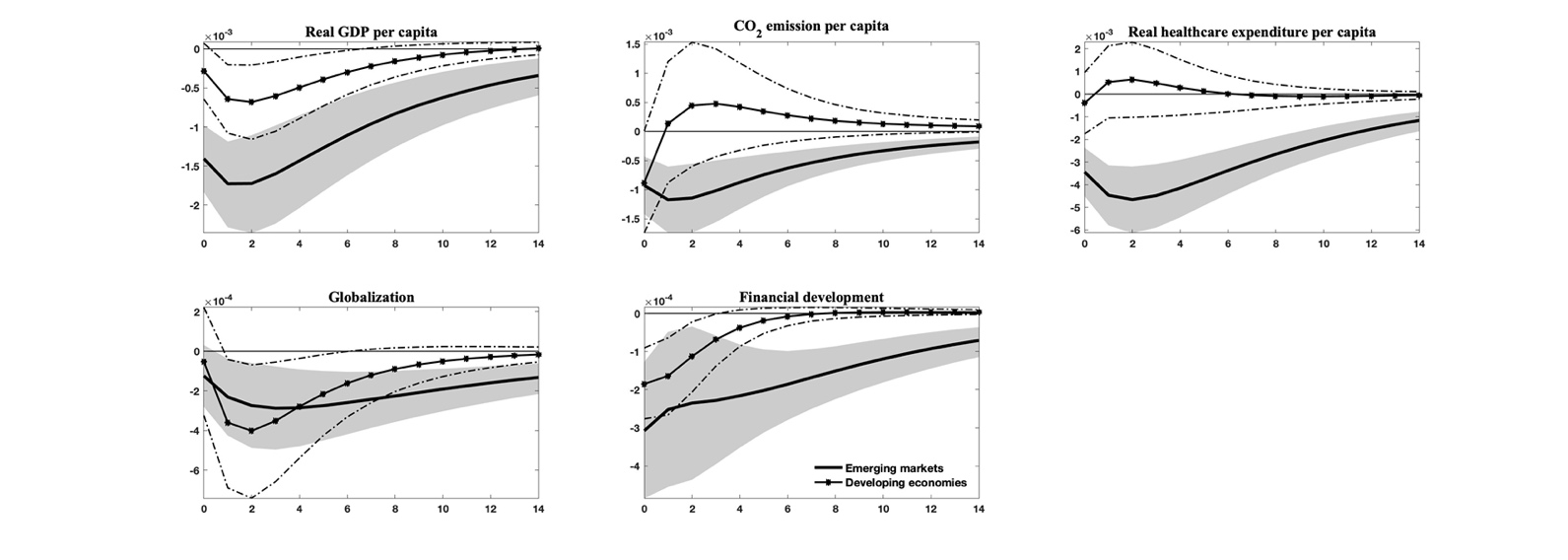


**Figure B4**

Selected impulse response functions following a 1% shock to economic uncertainty in emerging markets and developing countries. This figure illustrates the impact of economic uncertainty on several key factors, namely economic growth, environmental pollution, healthcare expenditure, financial development, and globalization, within the context of emerging markets and developing countries. Solid lines and solid asterisk-marked lines represent the median of the posterior estimates of the corresponding IRFs, while shaded areas and dash-dot lines denote 68% error bands constructed using the 16th and 84th percentiles of the posterior estimates of the corresponding IRFs.


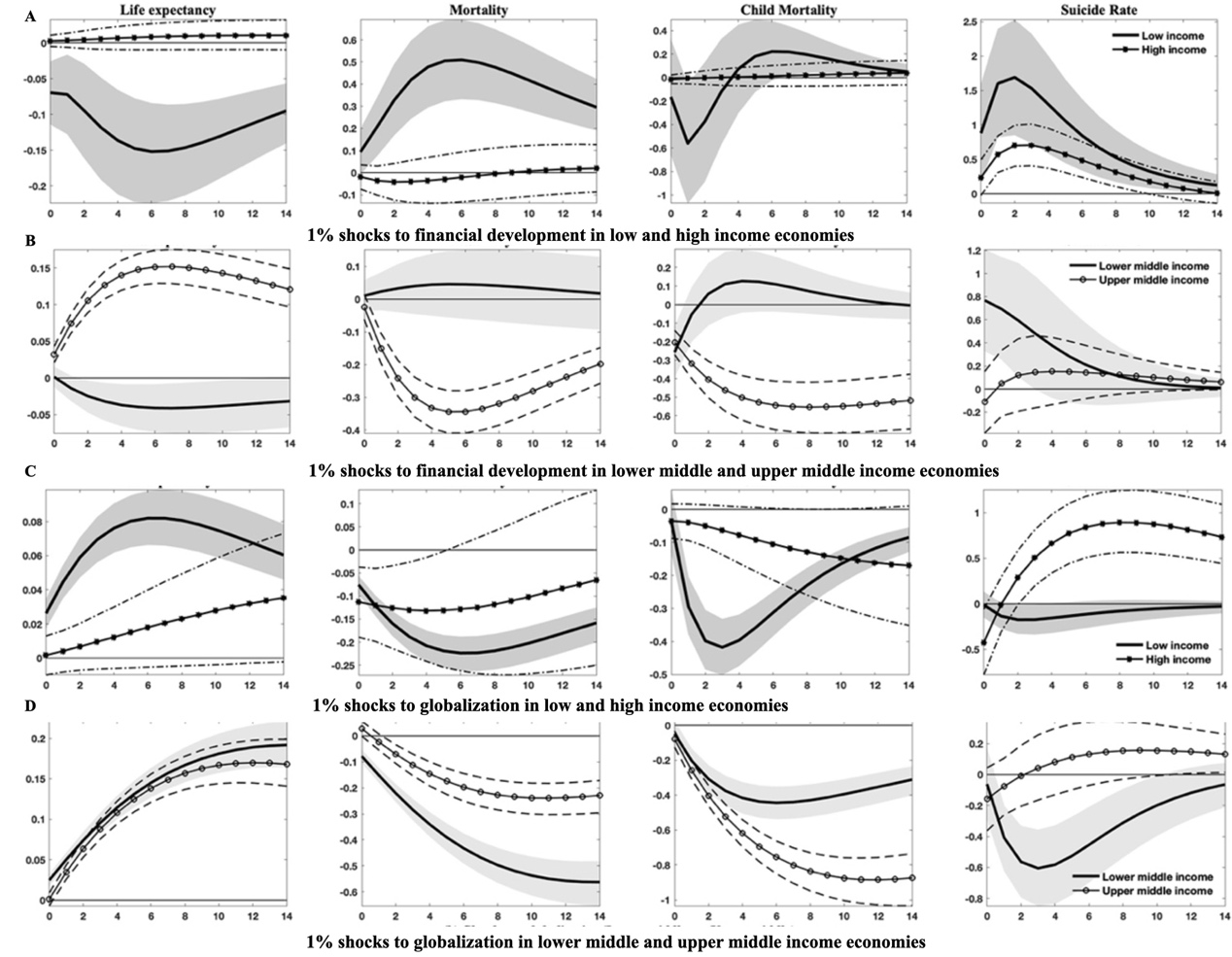


**Figure B5**

Health indicator responses to 1% shocks to financial development and globalization across economies at varying income levels. This figure reveals the differentiated trajectories of health indicators in the wake of advancements in financial development and globalization across countries with diverse income levels. Solid lines and solid dot/asterisk-marked lines denote the median of the posterior estimates of the corresponding IRFs, while shaded areas and dash(-dot) lines represent 68% error bands constructed from the 16th and 84th percentiles of the posterior estimates of the IRFs.


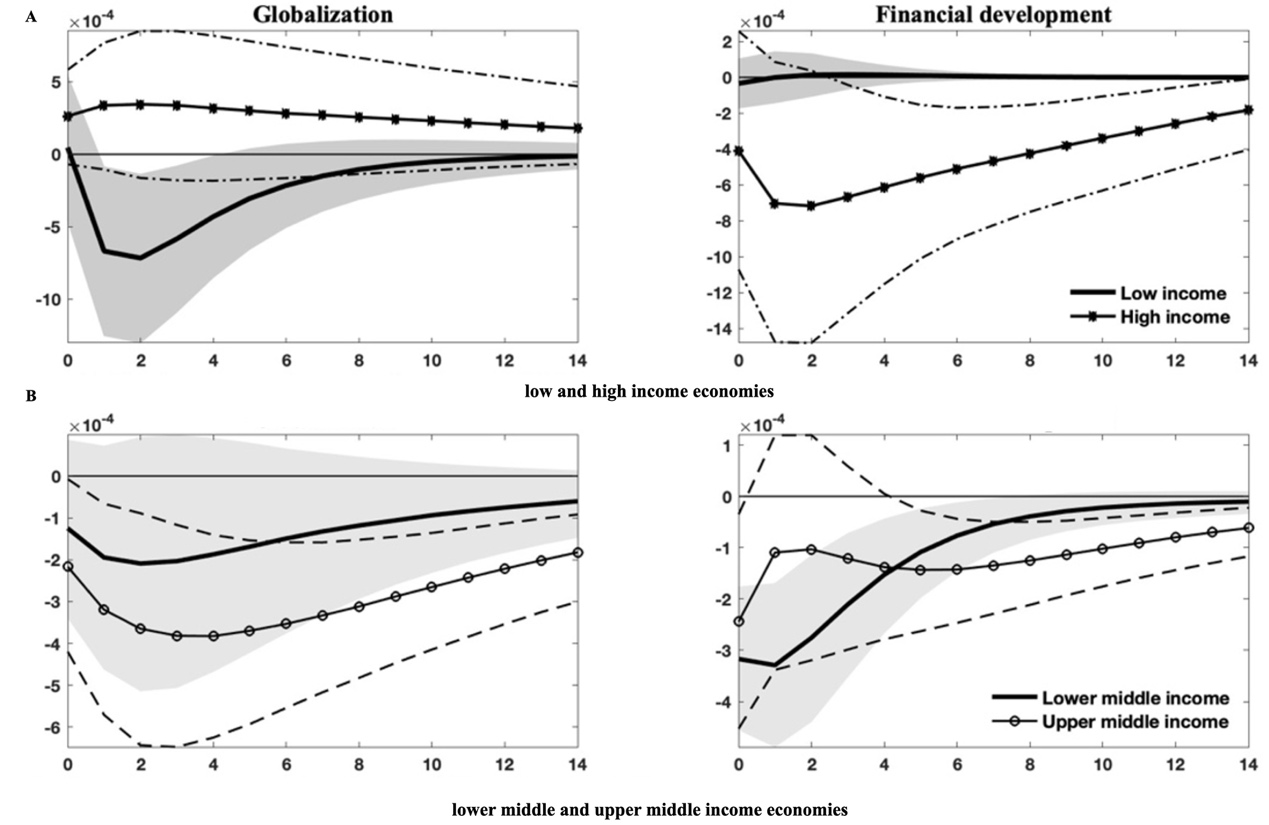


**Figure B6**

Responses of financial development and globalization to 1% shocks to economic uncertainty across economies of varying income levels. This figure elucidates the influence of economic uncertainty on financial development and globalization in countries with various income levels. Solid lines and solid dot/asterisk-marked lines depict the median of the posterior estimates of the corresponding IRFs, while shaded areas and dash(-dot) lines correspond to 68% error bands constructed using the 16th and 84th percentiles of the posterior estimates of the corresponding IRFs.

**References:**

1. Andrews DWK, Lu B. Consistent model and moment selection procedures for GMM estimation with application to dynamic panel data models. *J Econom*. (2001)101:123-164. doi:10.1016/S0304-4076(00)00077-4
